# Physicochemical stability and transfection efficiency of cationic amphiphilic copolymer/pDNA polyplexes for spinal cord injury repair

**DOI:** 10.1038/s41598-017-10982-y

**Published:** 2017-09-12

**Authors:** So-Jung Gwak, Christian Macks, Sooneon Bae, Noah Cecil, Jeoung Soo Lee

**Affiliations:** 1 Department of Bioengineering, Clemson University, Clemson, SC 29634 USA; 2 Department of Genetics and Biochemistry, Clemson University, Clemson, SC 29634 USA

## Abstract

Multiple age-related and injury-induced characteristics of the adult central nervous system (CNS) pose barriers to axonal regeneration and functional recovery following injury. *In situ* gene therapy is a promising approach to address the limited availability of growth-promoting biomolecules at CNS injury sites. The ultimate goal of our work is to develop, a cationic amphiphilic copolymer for simultaneous delivery of drug and therapeutic nucleic acids to promote axonal regeneration and plasticity after spinal cord injury. Previously, we reported the synthesis and characterization of a cationic amphiphilic copolymer, poly (lactide-co-glycolide)-graft-polyethylenimine (PgP) and its ability to efficiently transfect cells with pDNA in the presence of serum. We also demonstrated the efficacy of PgP as a therapeutic siRhoA carrier in a rat compression spinal cord injury model. In this work, we show that PgP/pDNA polyplexes provide improved stability in the presence of competing polyanions and nuclease protection in serum relative to conventional branched polyethylenimine control. PgP/pDNA polyplexes maintain bioactivity for transfection after lyophilization/reconstitution and during storage at 4 °C for up to 5 months, important features for commercial and clinical application. We also demonstrate that PgP/pDNA polyplexes loaded with a hydrophobic fluorescent dye are retained in local neural tissue for up to 5 days and that PgP can efficiently deliver pβ-Gal in a rat compression SCI model.

## Introduction

Spinal cord injury (SCI) leads to complex pathological changes that include neuronal and glial cell death and axonal demyelination and degeneration. Primary injury involves initial trauma to local tissue caused by bone fracture or compression of the spinal cord. The subsequent secondary injury cascade involves hypoxia, excitotoxicity, and inflammatory responses resulting in local apoptosis, secondary neuronal cell death, and cavity formation^1, 2^. SCI results in severe sensory and motor deficits due to the poor regenerative capacity of the adult spinal cord and has negative social, psychological and economic impacts on the patient’s life^3^ Currently, there is no effective pharmacological therapy.

Regeneration in the adult CNS is hindered by growth inhibitory molecules present in myelin and the glial scar, limited expression of growth promoting adhesive and trophic molecules, and age- and injury-related changes in neuronal biochemistry. Recently, gene therapy has received attention as a potential approach to increase expression of growth-promoting molecules such as neurotrophins^4–8^. While several viral vectors have demonstrated efficient therapeutic gene transfer after CNS injury, they often lack specificity and evoke immune reactions and inflammation^9–11^. Nonviral vectors such as cationic lipids or polymers carriers are being developed and explored for their potential to transfer genes into the CNS due to their ability to deliver genetic material without risk of viral protein introduction and immune activation. Lu *et al*. reported that a cationic liposome, DC-Chol, can successfully transfer pEGFP-GDNF into rat spinal cord cells after intraspinal injection and the expressed GDNF enhanced the axonal regeneration and functional recovery after SCI^7^. Takahashi *et al*. demonstrated that Bcl2 gene delivery by lipofectamine can prevent retrograde cell death and minimize atrophy in the injured spinal cord^12^. In another study, Gwak *et al*., reported that intraspinal injection of PLGA/DC-Chol nanospheres loaded with plasmid encoding vascular endothelial growth factor (VEGF) increased angiogenesis at the injury site, improved axonal regeneration, and locomotor function after SCI^4^. Despite the progress achieved in these studies, there remains a need for nonviral vectors with improved stability, increased transfection efficiency, and expanded capability for co-delivery of additional molecule.

Cationic polymeric micelle nanoparticle systems based on amphiphilic block or graft copolymers have been utilized as carriers to deliver drug/gene therapeutics because of their high loading capacity and unique disposition characteristics *in vivo* ^13–16^. The ultimate goal of our work is to develop cationic, amphiphilic polymeric micelle nanoparticles for simultaneous delivery of drugs and therapeutic nucleic acids (pDNAs, siRNAs, ODNs, and miRNAs) to promote axonal regeneration and plasticity. Toward this end, we recently reported the synthesis and characterization of poly (lactide-co-glycolide)-graft-polyethylenimine (PgP) and its ability to efficiently transfect pGFP in various neural cell lines and primary chick forebrain neurons in 10% serum condition *in vitro*, as well as in the normal rat spinal cord^17^. We have also shown that PgP can deliver siRNA targeting RhoA, a critical signaling pathway activated by multiple extracellular inhibitors of axonal regeneration, to the injured spinal cord and maintain RhoA knockdown for up to 4 weeks post-injection, reduce astrogliosis and cavitation, and increase axonal regeneration^18^. In this paper, we show that PgP/pDNA polyplexes remain stable and retain transfection activity after lyophilization as well as after storage for 6 months at 4 °C, important features for commercial and clinical application. We also demonstrate that hydrophobic dye-loaded PgP/pDNA polyplexes are retained in injured spinal cord for up to 5 days after intraspinal injection and that PgP can efficiently deliver pβ-Gal in a rat compression SCI model.

## Results

### Stability of PgP/pDNA polyplex

**Figure 1:**
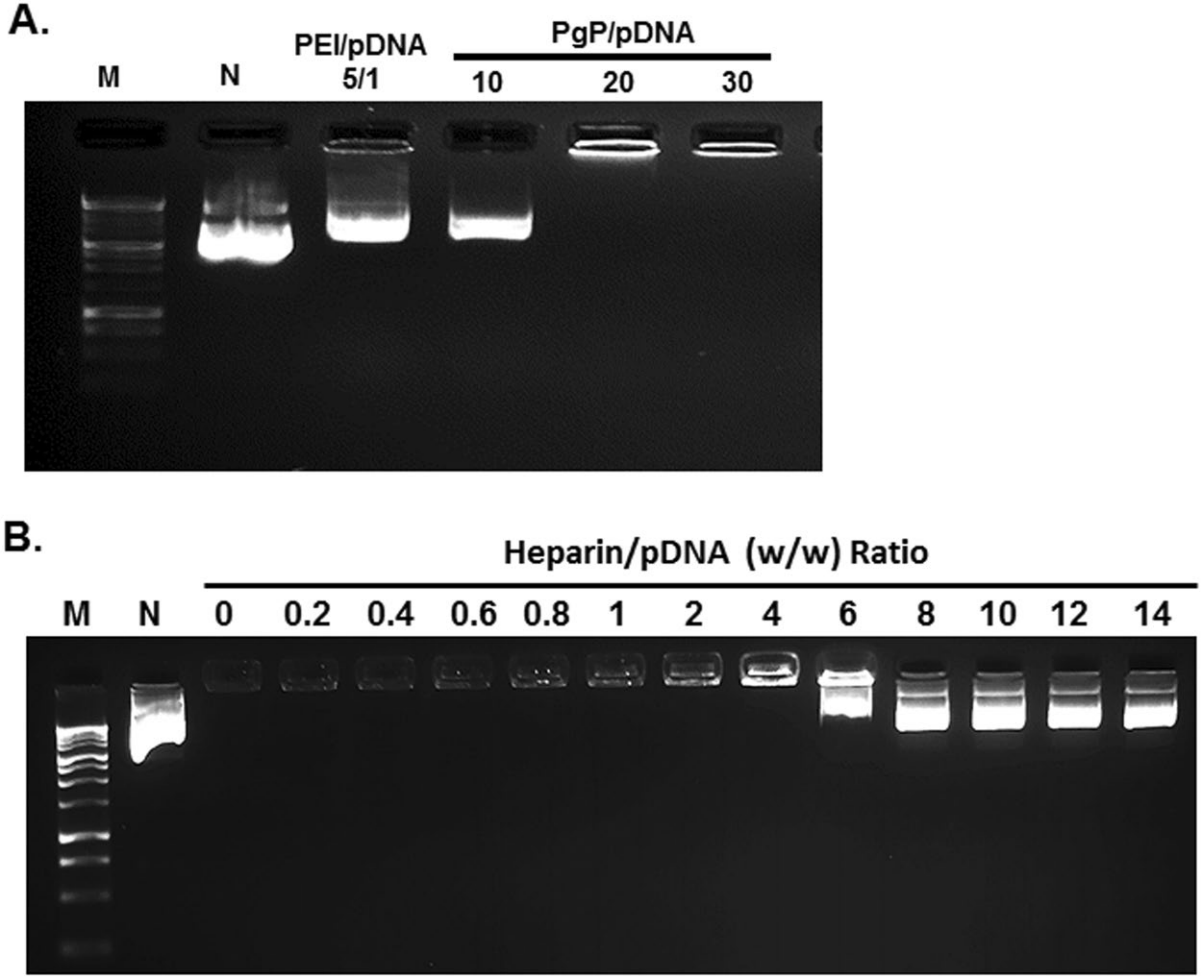
Heparin competition assay of PgP/pDNA polyplexes (2 µg pDNA). (**A**) PgP/pDNA polyplexes at varying N/P ratios and bPEI/pDNA polyplex at N/P ratio 5/1 were prepared and incubated in the presence of heparin (heparin/polyplexes ratio of 3/1 (w/w)) at 37 °C for 30 min. (**B**) PgP/pDNA polyplexes (2 µg pDNA) at N/P ratio of 30/1 incubated with solutions containing heparin at varying concentration (0–14 heparin/pDNA (w/w) ratios) at 37 °C for 30 min. M: Molecular marker, N: naked DNA.

**Figure 2:**
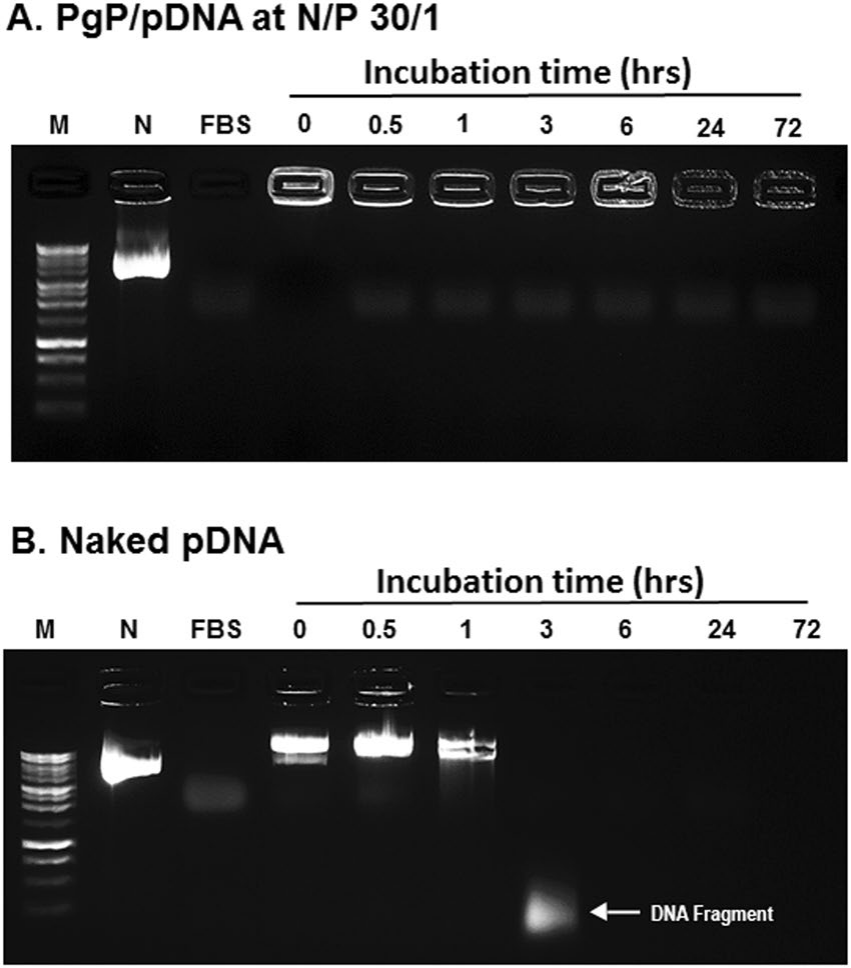
Stability of PgP/pDNA polyplexes (N/P ratio 30/1, 2 µg pDNA) after incubation in medium containing 50% serum at 37 °C. Molecular weight marker (M, Lane 1), naked DNA (N, lane 2), fetal bovine serum only (FBS, lane 3), and PgP/pDNA polyplexes at various time points during incubation in 50% serum for 0, 0.5, 1, 3, 6, 24, and 72 hrs (lane 4–10).

Previously, we have shown that PgP/pDNA forms stable polyelectrolyte complexes (polyplexes) at N/P ratios (nitrogen atoms in polymer to phosphate atoms in pDNA) of 10/1 and greater and achieves the highest transfection efficiency without significantly increased cytotoxicity at N/P of 30/1^17^. In these studies, we further examined polyplex integrity in the presence of competing polyanion (heparin) and serum nucleases. When polyplexes prepared at varying N/P ratios were incubated with 3/1 w/w (heparin/pDNA) ratio, PgP/pDNA at N/P ratio of 10/1 was dissociated, while polyplexes at N/P ratio 20/1 and 30/1 were stable (Fig. 1A). Polyplexes prepared using branched poly (ethylenimine) (bPEI, 25 kDa) with pDNA at N/P ratio of 5/1 were used as control and bPEI/pDNA polyplexes dissociated in the presence of heparin. To further characterize PgP/pDNA stability, polyplexes were prepared at N/P ratio of 30/1 and incubated in solutions with varying heparin concentration. PgP/pDNA polyplexes were stable in the presence of up to 4 heparin/pDNA (w/w) ratio and completely dissociated at ratios of 8 or higher (Fig. 1B). Polyplex integrity at N/P ratio of 30/1 in the presence of serum was evaluated after incubation in media containing 50% FBS. PgP/pDNA polyplexes remained stable for up to 3 days (Fig. 2A). However, naked pDNA was degraded by nucleases in the serum and undetectable after 3 hours incubation (Fig. 2B).

### Long-term shelf-life of PgP/pGFP polyplexes

**Figure 3:**
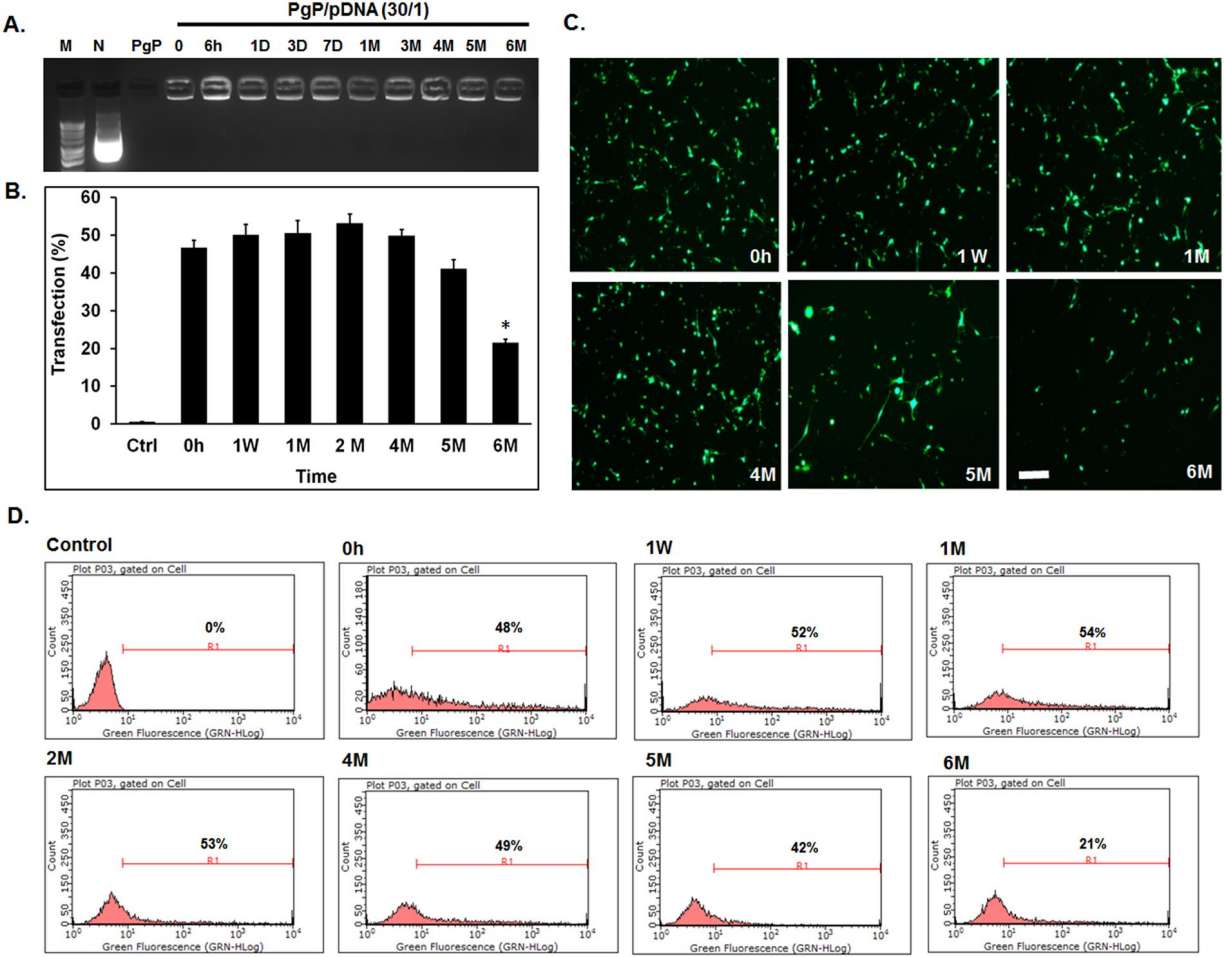
Long-term stability of PgP/pDNA polyplexes (N/P ratio of 30/1, 2 µg pDNA) in water at 4 °C. (**A**) Gel retardation assay of PgP/pDNA polyplexes stored at 4 °C for different time periods. Molecular weight marker (M, Lane 1), naked pDNA (N, lane 2), PgP only (lane 3), PgP/pDNA polyplexes at various time points during storage at 4 °C for 0, 6hrs, 1, 3, and 7 days, and 1, 3, 4, 5, and 6 months (lane 4–13). (**B**) Transfection efficacy of PgP/pGFP polyplexes stored at 4 °C in B35 cells in media containing 10% serum. *p < 0.05 compared with freshly prepared polyplexes. (**C**) Representative images and (**D**) Flow cytometry histogram of GFP + B35 cells at 2 days post-transfection with PgP/pGFP polyplexes stored at 4 °C. Scale bar indicates 200 µm. (Fresh: freshly prepared polyplexes, W: week, and M: Month).

To evaluate the long-term stability of PgP/pDNA polyplexes, polyplexes (N/P 30/1, 2 µg pGFP) were prepared and stored at 4 °C for 6 months. Gel electrophoresis analysis showed that polyplexes were stable up to 6 months at 4 °C (Fig. 3A). Transfection efficiency of PgP/pDNA polyplexes stored at 4 °C was maintained up to 5 months and not significantly different from freshly prepared PgP/pGFP polyplexes (~48%) (Fig. 3B and D). Polyplex transfection efficiency decreased after 6 months storage (~21%). Figure 3C and D shows representative images and flow cytometry histogram of GFP+ cells after transfection with PgP/pGFP polyplexes stored at 4 °C, respectively.

### Stability and transfection efficiency of lyophilized PgP/pDNA polyplexes

**Figure 4:**
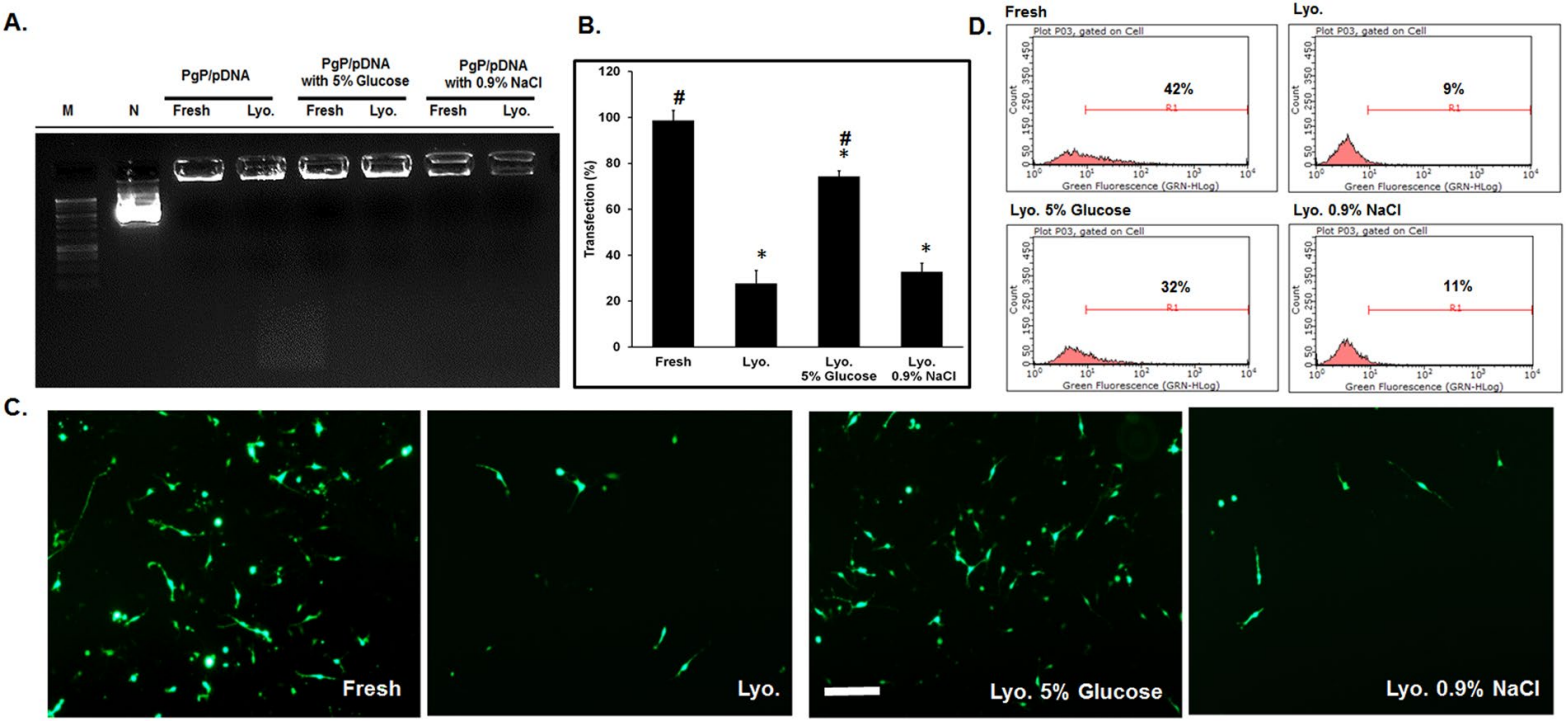
Stability and transfection efficiency of PgP/pGFP polyplexes after lyophilization. (**A**) Stability of PgP/pGFP polyplexes (N/P ratio 30/1) freshly prepared and after lyophilization alone or with the inclusion of cryoprotectants. Molecular weight maker (Lane 1), naked plasmid GFP (lane 2), fresh polyplexes (lane 3), reconstituted polyplexes after lyophilization (lane 4), fresh polyplexes with 5% glucose (lane 5), reconstituted polyplexes after lyophilization with 5% glucose (lane 6), fresh polyplexes with 0.9% NaCl (lane 7), reconstituted polyplexes after lyophilization with 0.9% NaCl (lane 8). (**B**) Transfection efficacy of freshly prepared and lyophilized PgP/pGFP polyplexes in B35 cells. *p < 0.05 compared with freshly prepared polyplexes. ^#^ *p* < 0.05 compared with lyophilized polyplexes without cryoprotectant. (**C**) Representative images and (**D**) flow cytometry histogram of GFP+ B35 cells at 2 days after transfection with PgP/pGFP polyplexes. Fresh: Freshly prepared polyplexes, Lyo.: Lyophilized PgP/pGFP, Lyo. 5% Glucose: Lyophilized PgP/pGFP with 5% glucose, Lyo. 0.9% NaCl: Lyophilized PgP/pGFP with 0.9% NaCl. Scale bar indicates 200 µm.

To evaluate the stability and transfection efficiency of PgP/pGFP polyplexes (N/P 30/1, 2 µg pGFP) after lyophilization with two different cryoprotectants, polyplexes were prepared in water and then lyophilized using either 5% glucose or 0.9% NaCl solution. Freshly prepared polyplexes without cryoprotectant were used as a control. Gel electrophoresis showed that polyplexes were stable after lyophilization under all conditions (Fig. 4A). PgP/pGFP polyplexes lyophilized from solutions containing 5% glucose maintained up to 83% transfection efficiency relative to freshly prepared polyplexes (Fig. 4B). In contrast, the transfection efficiency of polyplexes lyophilized without cryoprotectant or with 0.9% NaCl solution was reduced to 45.7% and 31.2%, respectively. Figure 4C and D show representative images and flow cytometry histograms of GFP+ cells after transfection with PgP/pGFP polyplexes lyophilized under various conditions.

### Cytotoxicity of PgP/pβ-gal polyplexes in normal spinal cord *in vivo*

**Figure 5:**
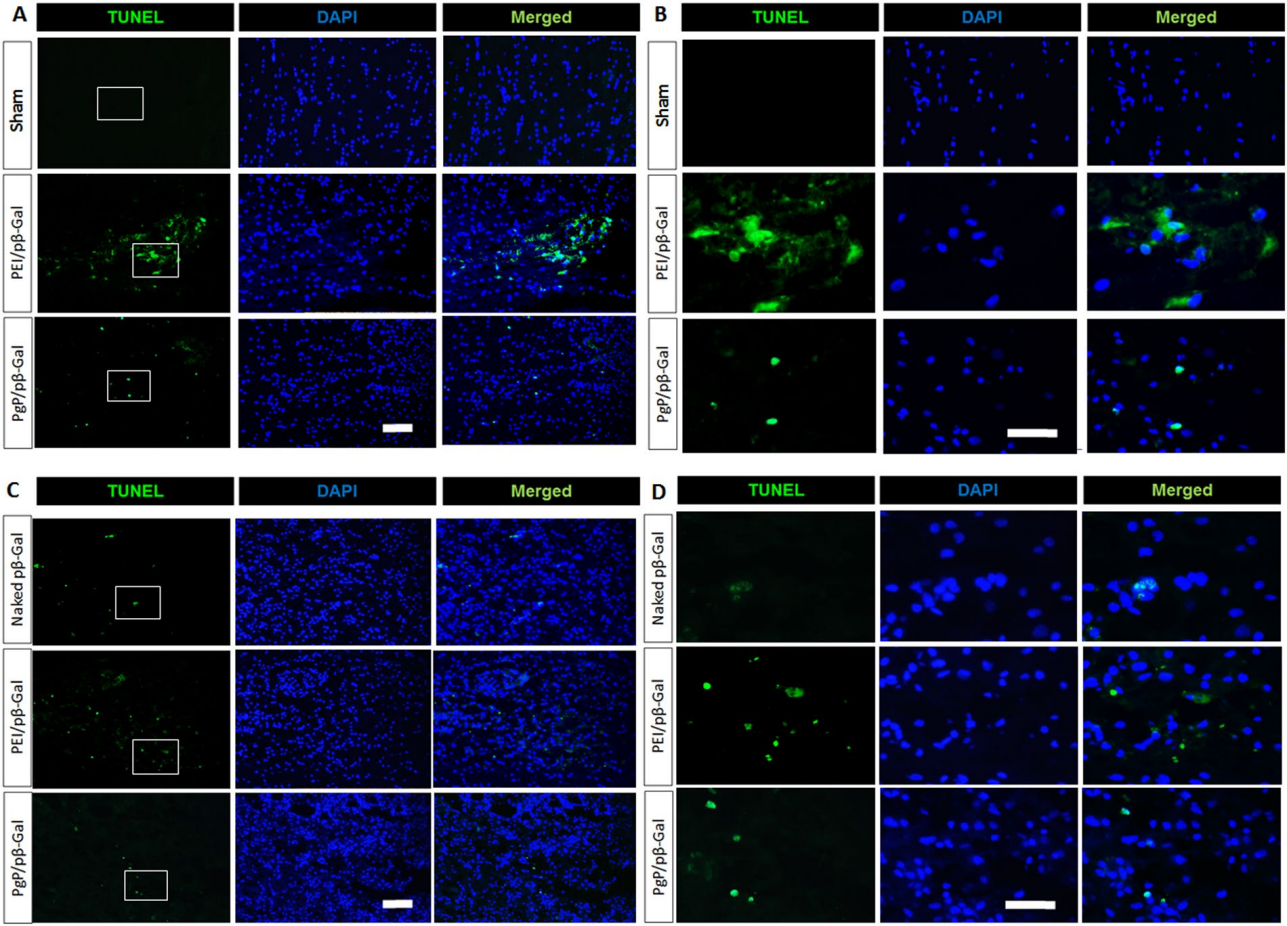
Cytotoxicity of PgP/pβ-Gal polyplexes at N/P ratio of 30/1 in normal rat spinal cord. At 2 and 7 days post-injection, rats were sacrificed and spinal cords explanted, embedded, sectioned, and toxicity was evaluated by TUNEL staining. Apoptotic cells were stained in green with blue DAPI nuclear counterstaining. (**A** and **B**) show representative images of TUNEL+ cells in spinal cord at 2 days post-injection (Top: sham control, Middle: bPEI/pβ-Gal polyplexes at N/P ratio of 5/1, Bottom: PgP/pβ-Gal polyplexes at N/P ratio of 30/1). (**C** and **D**) show representative images of TUNEL+ cells in spinal cord at 7 days post-injection (Top: naked pDNA, Middle: bPEI/pβ-Gal polyplexes at N/P ratio of 5/1, Bottom: PgP/pβ-Gal polyplexes at N/P ratio of 30/1), (**A** and **C**) Original magnification 200X (scale bar indicates 100 µm), (**B** and **D**) Enlarged images of highlighted interest region, original magnification 400X (scale bar indicates 50 µm).

To evaluate PgP as a non-viral gene carrier for SCI repair, we first tested the toxicity of PgP/pβ-gal polyplexes (N/P 30/1) relative to naked pβ-galactosidase (pβ-gal) or bPEI/pβ-gal (N/P 5/1). At 2 and 7 days post-injection, spinal cords were harvested, sectioned, and toxicity was evaluated by TUNEL staining. Figure 5 shows representative images of apoptotic (TUNEL+) and total cells (DAPI+ cells) in the normal spinal cord. We observed that the number of TUNEL+ cells was substantially lower after injection of PgP/pβ-gal than bPEI/pβ-gal polyplexes at both 2 days (Fig. 5A and B) and 7 days (Fig. 5C and D). However, both polyplexes showed more apoptotic cells than the sham control at 2 days-post injection and naked pDNA control at 7 days post-injection.

### Retention of DiR loaded PgP/pβ-gal polyplexes in SCI lesion

**Figure 6:**
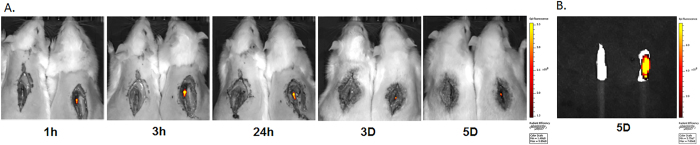
Retention of DiR-PgP/pDNA polyplexes (N/P ratio of 30/1) after local injection in SCI lesion site. (**A**) Visualization of DiR-PgP/pβ-gal polyplexes at 1, 3, 24 hours, and 3, and 5 days following injection by live animal fluorescence imaging system (IVIS). Left: Uninjected SCI animal (Control), Right: DiR-PgP/pβ-gal polyplexes injected animal (**B**) *Ex vivo* fluorescent imaging of DiR-PgP/pDNA polyplexes at 5 days post-injection by IVIS.

To visualize intraspinally injected polyplexes in the injured spinal cord, a hydrophobic fluorescent dye, 1, 1-dioctadecyl-3, 3, 3, 3-tetramethylindotricarbocyanine iodide (DiR) was loaded in PgP and then DiR-PgP/pDNA polyplexes (N/P ratio 30/1, 10 µg pDNA) were prepared and injected in the T9 spinal cord lesion site. Figure 6A shows representative images of injected polyplexes in the SCI lesion site at different post-injection time points. Each image shows an animal injected with DiR-PgP/pDNA polyplexes (Right) and uninjected control animal for comparison (Left). DiR-PgP/pDNA polyplexes were detectable for up to 5 days post-injection. Figure 6B shows a image from spinal cords explanted at 5 days post-injection.

### Beta-gal expression after local injection of PgP/pβ-gal in injured spinal cord

**Figure 7:**
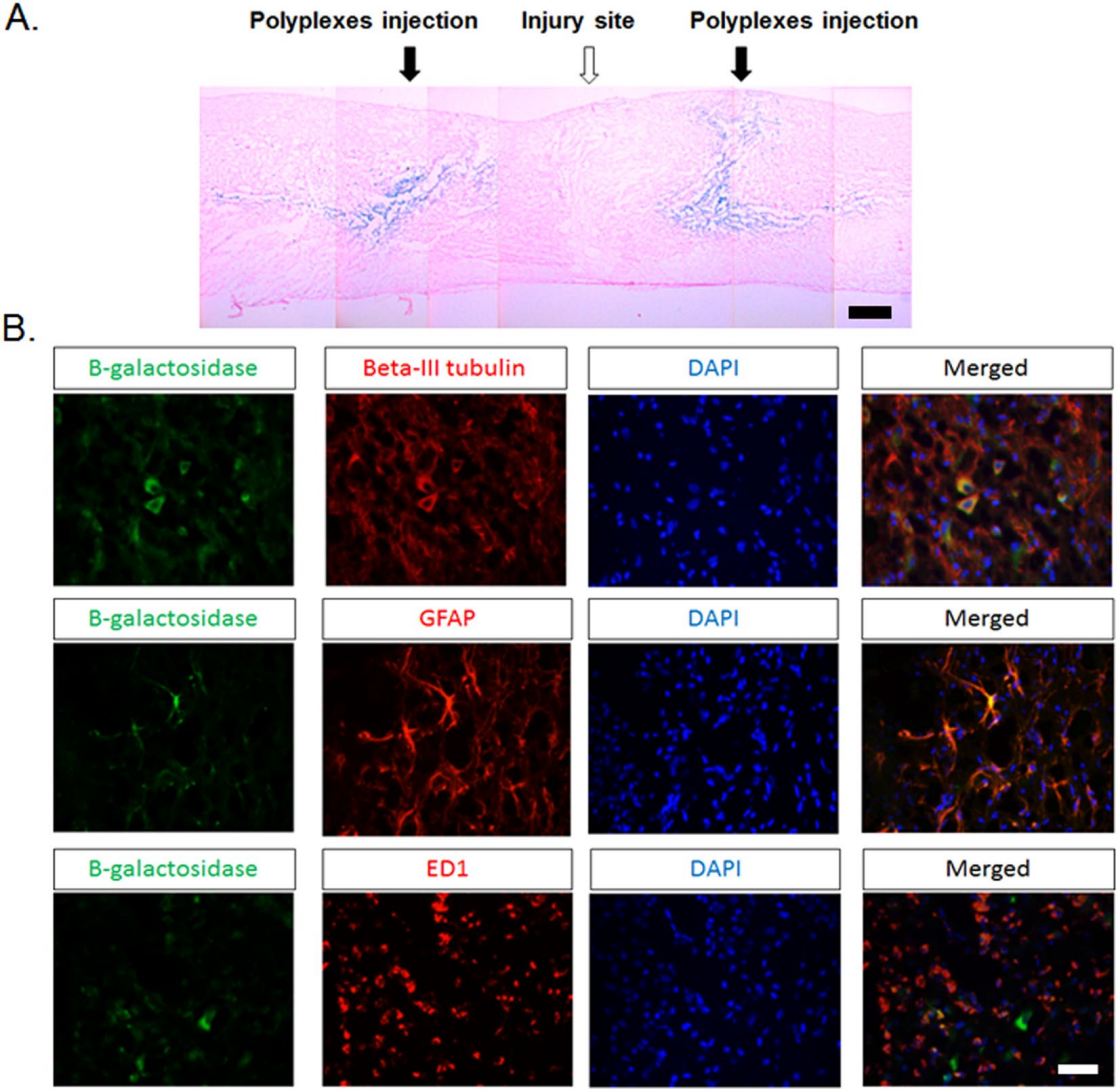
(**A**) Representative image of β-Gal expression (blue staining) in SCI model 7 days after injection of PgP/pβ-gal polyplexes at N/P ratio 30/1. Scale bar indicates 400 µm. (**B**) Double immunohistochemical staining for β-Gal+ cells (green) and beta-III tubulin (red, Left), GFAP (red, Middle) and ED-1 (red, Right) in SCI region. Merged images show co-localization of β-Gal+ cells and beta-III tubulin+ neurons, GFAP+ astrocytes, and ED-1+ microglia cells/infiltrated macrophages. Scale bar indicates 50 µm.

To evaluate PgP as a non-viral gene carrier in SCI repair, β-galactosidase expression was evaluated after injection of PgP/pβ-gal polyplexes (N/P 30/1, 10 µg). Figure 7A shows representative images of β-Gal+ cells stained in blue at injection site rostral and caudal to the lesion. We also used immunohistochemistry to identify the phenotype of β-Gal+ cells and found that these were predominantly beta-III tubulin+ neurons and GFAP+ astrocytes with a few ED-1+ activated microglia cells/infiltrated macrophages (Fig. 7B).

## Discussion

Many studies report gene delivery using cationic polymers as nonviral vectors for high transfection efficacy into neuronal cells *in vitro* ^19, 20^ and axonal regeneration and functional recovery after spinal cord injury^4^. bPEI has been one of the most widely applied non-viral gene delivery carriers, however, its potential for clinical therapy has been limited due to relatively high toxicity, low transfection efficiency in the presence of serum *in vitro* and aggregation with serum proteins in blood stream^21^. Several studies reported modification of PEI with hydrophobic groups and some showed that these derivatives achieved increased transfection efficiency with reduced toxicity in the presence of serum^22–24^, while the others showed lower transfection efficiency than the parent PEI in the presence of serum^25, 26^. In our previous study, we reported that our cationic, amphiphilic copolymer, PgP is capable of efficiently transfecting pDNA in the presence of 10% serum in various cell types including primary chick forebrain neuron cells as well as in the rat normal spinal cord *in vivo* ^17^. In this present work, we further evaluated the efficacy of PgP as a gene delivery carrier in a rat compression spinal cord injury model.

We first evaluated the stability of PgP/pDNA polyplexes by gel retardation after heparin competition assay and incubation in media containing 50% serum. PgP/pDNA at N/P 20/1 and 30/1 exhibited increased stability in the presence of a competing anionic macromolecule (heparin) relative to bPEI^27, 28^. We also observed that polyplexes prepared at N/P ratio of 30/1 were stable up to 3 days in the presence of 50% serum while naked pDNA was degraded by serum nucleases within 3 hours. Rapid degradation of naked pDNA exposed to serum has been previously reported by others. For example, Wang *et al*. found that naked pDNA was degraded in 3 hours and its band completely disappeared after 12 hours incubation in 50% serum^28^. Frickhofen *et al*. also reported that pDNA was completely degraded within 3 hours during storage in serum at room temperature^29^.

Preservation of bioactivity during long-term storage is another important challenge for the clinical translation of non-viral vectors. Previously, we showed that PgP can maintain stable complexes with siRNA up to 4 weeks at 4 °C^18^. In the present study, PgP/pDNA polyplexes showed physico-chemical stability and retained transfection efficiency after storage for up to 5 months at 4 °C. PgP/pDNA polyplexes also maintained their transfection efficiency (83% relative to freshly prepared samples) after lyophilization when glucose was used as a cryoprotectant. Several groups reported that lyophilization of polycation/pDNA complexes resulted a significant loss of gene transfection efficiency compared to freshly prepared complexes^30, 31^. Hahn *et al*. compared the activity of linear and branched PEI polyplexes after lyophilization and observed significant loss of activity for bPEI polyplexes that appeared to be related to changes in dissociation ability^32^. Mishra *et al*. reported a (PLGA)_2_-bPEI amphiphilic block copolymer that exhibited increased transfection efficiency after lyophilization/reconstitution relative to freshly prepared control, however, the overall transfection efficiency was lower than freshly prepared bPEI control^25^. Therefore, with respect to previous studies, PgP offers a bPEI derivative with significantly increased transfection efficiency relative to the parent polymer^17^ that can be preserved during prolonged storage in solution or after lyophilization/reconstitution.

To evaluate the feasibility of PgP as a non-viral gene carrier for the efficient treatment of SCI by gene therapy, we evaluated cytotoxicity, vector residence time in tissue post-injection, and *in vivo* transfection. In order to avoid confounding effects of injury on cell viability, we first evaluated PgP/pDNA cytotoxicity in the normal spinal cord. We found that PgP/pβ-Gal polyplexes (N/P ratio of 30/1) injected in a rat T9 normal spinal cord were less cytotoxic than bPEI/pβ-Gal polyplexes (5/1) by TUNEL assay.

In addition, the success of gene delivery carriers depends upon the choice of delivery route and residence time at the delivery site. In our previous study, we observed that intraspinally injected PgP/siRNA-Cy5 polyplexes were retained at the injury site up to 24 hours post-injection, while naked siRNA-Cy5 was undetectable after 6 hours, likely either as a result of degradation or diffusion away from injection site^18^. In this study, we used a hydrophobic dye (DiR) loaded into the micelle core to visualize PgP/pDNA polyplexes and evaluated longer time periods after injection of DiR-PgP/pDNA in SCI lesion sites. We observed that the intraspinally injected polyplexes were retained at the injection site up to 5 days.

Finally, we evaluated the efficacy of PgP as a pDNA carrier in a compression SCI model. pβ-gal was used to avoid potential artifacts originating from tissue autoflourescence. PgP/pβ-Gal polyplexes at N/P ratio of 30/1 showed substantial beta-gal expression in the injection site and surrounding neural tissue and histological analysis showed that the β-gal+ cells included neurons, astrocytes, and activated microglia cells/infiltrated macrophages. Several previous studies have reported successful therapeutic gene delivery by non-viral vectors in rat SCI model^7, 12^. Takahashi *et al*. reported that intraspinal administration of complexed plasmids encoding the Bcl-2 gene can prevent retrograde cell loss and reduce atrophy of axotomized red nucleus and Clarke’s nucleus neurons in rat hemisection spinal cord injury model^12^. In another study, Lu *et al*. showed that plasmids encoding GDNF delivered by DC-Chol liposome promoted axonal regeneration and enhanced locomotion function recovery in rat compression spinal cord injury model^7^. Therefore, we believe that PgP can be an effective carrier for pDNA encoding therapeutic genes to promote recovery from SCI. In addition to gene delivery, another unique feature is that PgP can be an efficient carrier for hydrophobic drugs, enabling combinatorial therapies using drugs targeting the inflammatory response during secondary injury or neuronal intracellular signaling pathways.

## Conclusion

In this study, we demonstrate that the cationic, amphiphilic copolymer PgP can form polyplexes with pDNA that remain stable in the presence of competing polyanions and provide protection from serum nucleases. In addition, PgP/pDNA polyplexes can be stored for up to 4 months at 4 °C and maintain their transfection efficiency after lyophilization/reconstitution, important features for commercial and clinical application. We also demonstrate that PgP polyplexes injected intraspinally remain present in the local tissue for up to 5 days and achieve substantial beta-gal expression in the injured spinal cord and surrounding neural tissues. In the future, we will evaluate the efficacy of PgP as a therapeutic gene delivery carrier in preclinical animal models of SCI, traumatic brain injury, stroke, and other neurodegenerative diseases such as Alzheimer’s Disease, Parkinson’s diseases, Huntington’s Diseases, and Amyotrophic Lateral Sclerosis.

## Materials and Methods

### Plasmid amplification and purification

Plasmids encoding the Monster Green Fluorescent Protein (phMGFP Vector, pGFP, Promega, Medison.WI) and beta-galactosidase (pSV40-pβGal, pβGal, Promega) were transformed into *Escherichia coli* DH5α (Invitrogen, Carlsbad, CA) and amplified in LB medium (Sigma-Aldrich, St. Louis, MO) at 37 °C overnight with shaking at 250 rpm. pGFP and pβGal were isolated using the Endofree Maxi Plasmid purification kit (Qiagen, Valencia, CA) according to the manufacturer’s instructions. The quantity and quality of pDNA were determined using Biotek Take 3 microplate reader by the absorbance at 260/280 nm (BioTek, Synergy HT).

### PgP Synthesis and characterization

Cationic, amphiphilic copolymer PgP (poly(lactide-co-glycolide)–g-polyethylenimine) was synthesized using branched polyethylenimine (bPEI) (MW 25 kDa, Sigma) and poly (lactide-co-glycolide) (PLGA 4 kDa, 50:50, Durect Corporation, Pelham, AL) with a carboxylic end group as previously described^17^. Briefly, the carboxylic acid group of PLGA was activated by *N*-hydroxysuccinimide (NHS) and *N,N’*-dicyclohexylcarbodiimide (DCC). The activated PLGA was conjugated to primary amine groups of bPEI. PgP was purified by dialysis against deionized water, centrifuged, filtered and lyophilized. The structure and molecular weight of PgP was determined by ^1^H- NMR (300 MHz, Bruker) using D_2_O as a solvent and gel permeation chromatography. These studies indicated that approximately 3 PLGA polymers were grafted to each bPEI and the molecular weight of PgP was determined as approximately 38,168 by GPC using dextran standards.

### Stability of PgP/pDNA polyplexes by heparin competition assay

To evaluate the stability of polyplexes, we first tested the effect of N/P ratio on the stability of PgP/pDNA polyplexes by heparin competition assay. PgP/pDNA polyplexes were prepared at N/P ratios 10, 20, and 30. Briefly, polyplexes were prepared by adding 50 μl DNA (2 μg pDNA) solution into 50 μl PgP solutions containing varying amounts of PgP with gentle mixing and then the solutions were incubated for 30 min at 37 °C. bPEI/pDNA (2 μg pDNA) at N/P ratio 5/1 was prepared and used as a positive control. Polyplexes were incubated with heparin solution (3/1 w/w ratio, heparin/pDNA) at 37 °C for 30 minutes and then the samples were immediately electrophoresed on a 1% (w/v) agarose gel with SYBR™ Safe™ DNA Gel Stain (Invitrogen) for 45 min at 80 V. The gel was imaged on a UV illuminator (Chemidoc-It^2^, UVP) to visualize polyplex retention and naked pDNA migration. Next, we evaluated the stability of PgP/pDNA (2 μg pDNA) polyplexes at N/P ratio of 30/1 (optimal N/P ratio from our previous study) after exposure to heparin/pDNA at 0 to 14 w/w ratio at 37 °C for 30 min and then the samples were immediately analyzed by agarose gel electrophoresis.

### Stability of PgP/pDNA polyplexes in media containing 50% serum

To simulate *in vivo* conditions, the ability of PgP to protect pDNA from nucleases in the serum was tested in media containing 50% serum. PgP/pGFP (2 μg pDNA) at N/P ratio of 30/1 was prepared and incubated in media containing 50% serum for 72 hours at 37 °C. Naked pDNA (2 μg pDNA) was also tested for comparison. At pre-determined time points, polyplexes or naked pDNA were collected and analyzed by agarose gel electrophoresis.

### Long-term shelf-life and transfection efficiency of stored PgP/pDNA polyplexes

To evaluate the long-term stability of polyplexes during storage, PgP/pDNA (2 μg pDNA) polyplexes at N/P ratio of 30/1 were prepared in water and stored at 4 °C for 6 months. At pre-determined time points, polyplexes were collected and the stability of polyplexes was analyzed by agarose gel electrophoresis and the transfection efficiency was evaluated in rat neuroblastoma (B35) cells. Neuroblastoma (B35, 8 × 10^4^ cells/well) cells were seeded in 24-well plates using DMEM/F12 supplemented with 10% FBS and 100 IU/ml penicillin/100 µg/ml streptomycin. After overnight incubation, the cells were washed twice with fresh media containing 10% serum. The cells were transfected with PgP/pDNA polyplexes stored at 4 °C and incubated for 24 hours. At 24 hours post-transfection, the media were replaced by fresh medium containing 10% FBS and transfected cells were incubated an additional 24 hours. Freshly prepared PgP/pGFP polyplexes (2 µg DNA, N/P 30/1) were used as a control. GFP expression was measured by flow cytometry (Guava easyCyte, Millipore) and imaged using an inverted epifluorescent microscope (Zeiss Axiovert 200, Göttingen, Germany).

### Stability and transfection efficiency of lyophilized PgP/pGFP polyplexes

To evaluate stability of PgP/pGFP polyplexes after lyophilization, PgP/pGFP polyplexes (2 µg DNA) at N/P ratio of 30/1 were prepared and frozen in solutions containing 5% glucose or 0.9% NaCl at −80 °C overnight followed by lyophilization. Lyophilized polyplexes were reconstituted with distilled water. Freshly prepared PgP/pGFP polyplexes at N/P ratio of 30/1 were used for comparison. The stability of lyophilized polyplexes was analyzed by agarose gel electrophoresis and the transfection efficiency evaluated in B35 cells as described above.

### Cytotoxicity of PgP/pβ-gal polyplexes in rat normal spinal cord *in vivo*

The toxicity of PgP/pβ-Gal polyplexes was evaluated after injection of polyplexes in normal rat spinal cord. All surgical procedures and postoperative care were conducted according to NIH guidelines for the care and use of laboratory animal (NIH publication No. 86–23, revised 1996) and under the supervision of the Clemson University Animal Research Committee (Approved animal protocol no. AUP 2014-012). Sprague Dawley rats (male, 200 gm) were anesthetized with isoflurane gas. After shaving their back, laminectomy was performed and T9 spinous processes were removed using orthopedic bone cutter and spinal cord was exposed. 9 rats were divided into 3 groups (n = 3/group). Group 1: 20 µl of PgP/pβ-Gal at N/P ratio of 30/1 (10 µg, pβ-Gal), Group 2: bPEI/pβ-Gal polyplexes at N/P ratio of 5/1 (10 µg, pβ-Gal), and Group 3: 20 µl of naked pβ-Gal (10 µg). The samples were injected using a 26 G Hamilton syringe (HAMILTON®, Reno, NV, USA) into the T9 normal spinal cord. At 2 and F7 days post-injection, the animals were anesthetized by isoflurane gas and sacrificed via cardiac perfusion with 4% paraformaldehyde solution (pH 7.4; Merck, Germany). The retrieved spinal cords were fixed with 4% paraformaldehyde solution and embedded into Tissue-Tek® O.C.T compound (Sakura Finetek USA Inc, CA) on liquid nitrogen and 10 µm thick sections cut longitudinally and mouted on positively charged glass slides. The sections were stained by TUNEL staining using the ApopTag Plus Fluorescein In situ Apoptosis Detection kit (Chemicon International. Temecula, CA) and nuclei were counterstained using DAPI. The stained sections were imaged using an inverted epifluorescent microscope (Zeiss Axiovert 200, Göttingen, Germany).

### Generation of rat compression spinal cord injury model

Sprague Dawley rats (male, 200 gm) were anesthetized with isoflurane gas. After shaving their back, laminectomy was performed and then the T9 spinous processes were removed using orthopedic bone cutter. The compression SCI was performed as previously described by Gwak *et al*.^4, 18^. The exposed T8~T9 spinal cord was compressed by a vascular clip (75 g force) for 10 min. Following injury, the paraspinal muscles were closed with 3–0 silk suture and the skin was closed with surgical clips. After surgery, animals were warmed by heating blanket for recovery. For 1 weeks after surgery, animals received cefazolin antibiotic (40 mg/Kg, Hikma Farmaceutic) and buprenorphine analgesic (0.01 mg/kg, Hospira Inc) and bladders were manually expressed three times daily.

### Retention of DiR loaded PgP/pβ-gal in the SCI lesion site

To evaluate the retention of injected polyplexes in the SCI lesion site, hydrophobic fluorescent dye, 1,1-dioctadecyl-3,3,3,3-tetramethyl indo tricarbocyanine iodide (DiR, PromoCell GmbH, Germany) was loaded in the hydrophobic core of PgP by solvent evaporation method. Briefly, DiR dye was dissolved in ethanol and DiR dye solution was added into PgP micelle (1 mg/ml) aqueous solution and then incubated for 4 hrs at room temperature under constant stirring. After loading, DiR-PgP solution was further incubated overnight to allow ethanol evaporation. The DiR-PgP was filtered with 0.2 µm syringe filter to remove unloaded DiR dye. DiR-PgP/pβ-Gal (10 µg pDNA) polyplexes at N/P ratio 30/1 were prepared by mixing pβ-Gal solution with DiR-PgP. The spinal cord injury was generated as described above and10 µl of DiR-PgP/pβ-Gal polyplexes were injected into the each side of SCI lesion site using Hamilton syringe (G26). The retention and distribution of DiR-PgP/pβ-gal polyplexes were assessed immediately following injection by live animal fluorescence imaging system (IVIS Luminar XR, Caliper Life Sciences) at 1, 3, 24 hours, and 3, and 5 days. At 5 days post-injection, spinal cords (0.5 cm-long piece from the center of the injury) were harvested and imaged by live animal fluorescence imaging system (IVIS Luminar XR, Caliper Life Sciences).

### β-Gal expression after injection of PgP/pβ-Gal polyplexes *in SCI lesion*

To evaluate PgP as a non-viral gene carrier in CNS injury *in vivo*, compression SCI was generated as described above. In this study, we used plasmid encoding β-Galactosidase (pSV40-β-gal: pβ-gal, Promega) to avoid potential interference of tissue autofluoresecence with GFP analysis. 10 µl of PgP/pβ-gal polyplexes (N/P ratio 30/1, 10 µg pβ-gal/20 µl polyplexes) were injected into the each side of SCI lesion site using Hamilton syringe (G26). At 7 days post-injection, animals were sacrificed via cardiac perfusion with 4% paraformaldehyde solution. The retrieved spinal cords were fixed with 4% paraformaldehyde solution and embedded into Tissue-Tek® O.C.T compound (Sakura Finetek USA Inc, CA) on liquid nitrogen and 10 µm thick sections cut longitudinally and mouted on positively charged glass slides. To evaluate β-Gal expression in spinal cord injury site, sections were stained using a β-Gal staining kit (Life Technologies) to detect β-Gal positive (β-Gal+) cells. To evaluate the phenotype of β-Gal+ cells, sections were double immuno-stained using antibodies against β-Gal (mouse anti- β-Gal, chick anti- β-Gal, Abcam) and beta III tubulin for neurons (Abcam), GFAP for astrocytes (rabbit anti-GFAP, Abcam) or ED-1 for microglia (mouse anti-monocytes/macrophases, Millipore), respectively. Cy3-conjugated anti-rabbit IgG and FITC-conjugated anti-mouse IgG secondary antibodies (Jackson Immunoresearch Laboratories) were applied and nuclei were counterstained using DAPI. The stained sections were digitally imaged using an inverted epifluorescent microscope (Zeiss Axiovert 200,Göttingen, Germany) and Nikon AZ100 (Nikon, Japan).

### Statistical analysis

Quantitative data are presented as the mean ± standard deviation. The statistical significance was analyzed between groups by a one-way ANOVA. A *p*-value less than 0.05 was considered significant.
